# Lymphocyte Modulation with FTY720 Improves Hemorrhagic Shock Survival in Swine

**DOI:** 10.1371/journal.pone.0034224

**Published:** 2012-04-30

**Authors:** Jason S. Hawksworth, J. Christopher Graybill, Trevor S. Brown, Shannon M. Wallace, Thomas A. Davis, Doug K. Tadaki, Eric A. Elster

**Affiliations:** 1 Regenerative Medicine Department, Operational and Undersea Medicine Directorate, Naval Medical Research Center, Silver Spring, Maryland, United States of America; 2 Department of Surgery, Walter Reed National Military Medical Center, Bethesda, Maryland, United States of America; 3 Uniformed Services University of Health Sciences, Bethesda, Maryland, United States of America; 4 Department of Diagnostic Pathology, Walter Reed Army Institute of Research, Silver Spring, Maryland, United States of America; Heart Center Munich, Germany

## Abstract

The inflammatory response to severe traumatic injury results in significant morbidity and mortality. Lymphocytes have recently been identified as critical mediators of the early innate immune response to ischemia-reperfusion injury. Experimental manipulation of lymphocytes following hemorrhagic shock may prevent secondary immunologic injury in surgical and trauma patients. The objective of this study is to evaluate the lymphocyte sequestration agent FTY720 as an immunomodulator following experimental hemorrhagic shock in a swine liver injury model. Yorkshire swine were anesthetized and underwent a grade III liver injury with uncontrolled hemorrhage to induce hemorrhagic shock. Experimental groups were treated with a lymphocyte sequestration agent, FTY720, (n = 9) and compared to a vehicle control group (n = 9). Animals were observed over a 3 day survival period after hemorrhage. Circulating total leukocyte and neutrophil counts were measured. Central lymphocytes were evaluated with mesenteric lymph node and spleen immunohistochemistry (IHC) staining for CD3. Lung tissue infiltrating neutrophils were analyzed with myeloperoxidase (MPO) IHC staining. Relevant immune-related gene expression from liver tissue was quantified using RT-PCR. The overall survival was 22.2% in the vehicle control and 66.7% in the FTY720 groups (p = 0.081), and reperfusion survival (period after hemorrhage) was 25% in the vehicle control and 75% in the FTY720 groups (p = 0.047). CD3^+^ lymphocytes were significantly increased in mesenteric lymph nodes and spleen in the FTY720 group compared to vehicle control, indicating central lymphocyte sequestration. Lymphocyte disruption significantly decreased circulating and lung tissue infiltrating neutrophils, and decreased expression of liver immune-related gene expression in the FTY720 treated group. There were no observed infectious or wound healing complications. Lymphocyte sequestration with FTY720 improves survival in experimental hemorrhagic shock using a porcine liver injury model. These results support a novel and clinically relevant lymphocyte immunomodulation strategy to ameliorate secondary immune injury in hemorrhagic shock.

## Introduction

The immune system has evolved to respond to localized injury and infection. Severe trauma and hemorrhagic shock result in systemic activation of the immune system, initiating an inappropriate inflammatory response that leads to secondary host ischemia reperfusion injury. Systemic inflammatory intensification following injury can progress to acute respiratory distress syndrome, systemic inflammatory response syndrome, and multiple organ dysfunction syndrome [1]. Therapeutic manipulation of the immunologic response to severe trauma may reduce associated morbidity and mortality.

Lymphocytes are major components of adaptive immunity and influence late immune dysfunction following severe injury [2], [3], [4]. Lymphocytes have also recently been identified as critical mediators of the early innate immune response to ischemia-reperfusion injury (IRI). Compelling pre-clinical investigation has established the innate role of lymphocytes in renal [5], gut [6], and liver [7], [8] IRI. In a renal IRI model, genetically engineered mice deficient in both CD4^+^ and CD8^+^ lymphocytes had substantially less kidney dysfunction after renal ischemia than did wild-type control mice [5]. Interestingly, mice deficient in CD4^+^ and CD8^+^ lymphocytes demonstrated less tissue neutrophil infiltration, suggesting that lymphocytes orchestrate cell-mediated innate responses to ischemia. Furthermore, lymphocytes have been shown to rapidly accumulate in target organs following ischemia and may represent very early cellular mediators of reperfusion injury [9]. Thereby, immunomodulation of lymphocytes may offer a novel approach to attenuate very early detrimental immune responses to severe traumatic injury.

The innate role of lymphocytes offers the potential for targeting lymphocytes in the setting of severe injury and hemorrhagic shock. FTY720, an immunomodulator recently approved for the treatment of multiple sclerosis, sequestrates lymphocytes to secondary lymphoid organs and reduces circulatory lymphocytes by targeting receptors for sphingosine 1-phosphate (S1P) [10], [11], [12]. Disruption of lymphocyte trafficking with FTY720 may also offer a therapeutic strategy for reperfusion injury associated with hemorrhagic shock.

In this study, we demonstrate that lymphocyte sequestration with FTY720 significantly improves reperfusion survival in a large animal hemorrhagic shock model. FTY720 resulted in sequestration of central lymphocytes and appeared to attenuate innate cellular and molecular activation following hemorrhagic shock. Lymphocyte immunomodulation strategies offer an exciting and widely applicable approach towards abrogating immune mediated reperfusion injury in trauma and surgical patients.

## Methods

### Animal Preparation

The experiments reported herein were conducted in compliance with the Animal Welfare Act and in accordance to the principles set forth in the “Guide for the Care and Use of Laboratory Animals,” Institute of Laboratory Animals Resources, National Research Council, National Academy Press, 1996. The study was approved by the National Medical Research Center Institutional Animal Care and Use Committee (IACUC, protocol KO05-06) and all procedures were performed in animal facilities approved by the Association for Assessment and Accreditation for Laboratory Animal Care International (AAALAC).

Male and female 3–12 month Yorkshire (*Sus scrofa domestica*) swine weighing 25–35 kg were acquired from ABI Farms (Donsboro, PA). Feed was withheld 12 hours before surgery. Prior to surgery, animals were sedated and anesthesia induced with intramuscular ketamine hydrochloride (12–20 mg/kg) and xylazine (2.2 mg/kg) as well as atropine sulfate (0.05 mg/kg) to decrease tracheal secretions. Mask ventilation with Isoflurane (5.0%) was used to facilitate endotracheal intubation. Animals were ventilated (Ohmeda 7800 series ventilator, Datex, Madison, WI) at 12–15 breaths/min and tidal volume 10 mL/kg. Anesthesia was maintained with Isoflurane (1.5–2.5%) in 21–25% O_2_.

Following adequate anesthesia the right external and internal jugular veins and carotid artery were isolated. A 9Fr dual-lumen, tunneled Hickman catheter was placed in the external jugular vein for drug infusion and blood collection during the survival period. A 9Fr introducer sheath was placed in the internal jugular vein and a 7.5Fr pulmonary artery catheter (PAC; Edwards Life Sciences, Irvine, CA) was inserted for continuous hemodynamic monitoring. An 18 G Angiocath was placed in the carotid artery and mean arterial pressure (MAP) was continuously transduced. A midline laparotomy was performed to expose the liver and isolate the left lateral lobe. Urine was collected via bladder catheterization. Rectal temperature was monitored continuously. Normothermia (37°C) was maintained with a warming device (Model 505, Bair Hugger, Augustine Medical, Eden Prairie, MN).

### Liver Injury and Resuscitation

**Figure 1:**
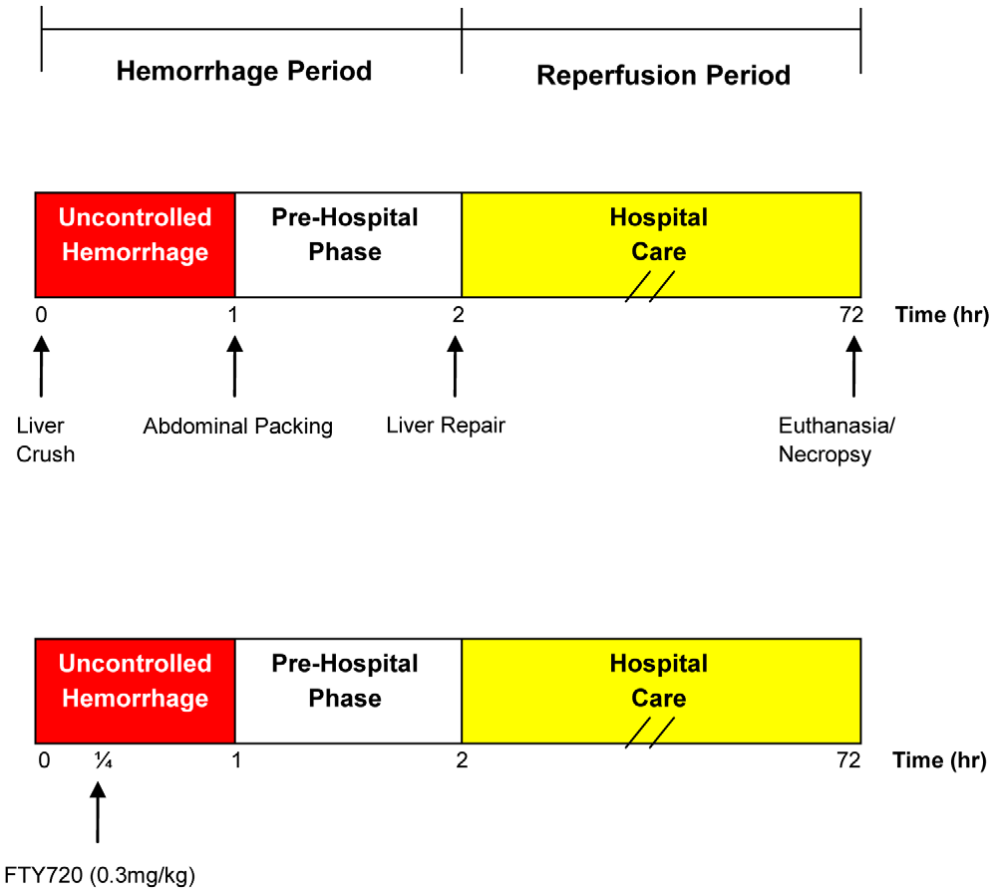
Experimental design. Liver injury was initiated at t = 0. Uncontrolled hemorrhage occurred until 1 hour, at which time the abdomen was packed and temporarily closed (pre-hospital phase). The animal was observed until 2 hours when hospital care was initiated. The liver was repaired and the abdomen definitively closed. The animal was then observed for a total of 72 hours. Blood transfusion was administered as indicated. Necropsy was performed when the animal expired or at 72 hours following euthanasia. The uncontrolled hemorrhage and pre-hospital phases were considered the hemorrhage period, while the hospital care phase was considered the reperfusion period. In the experimental group, FTY720 (0.3 mg/kg diluted in 250 mL of NS)) was administered 15 minutes following liver injury. Vehicle controls were treated with 250 mL of NS.

All animal groups underwent a standardized liver injury and resuscitation protocol. (Figure 1). A reproducible liver injury was created by placing a ring clamp over the left lower lobe, 50% in width and 3.5–5 mm from the apex, adjusting for the relative size of the animal. The clamp was closed and a #10 blade was used to lacerate the lobe from the top of the clamp through the remaining width. The liver injury denoted the start of the pre-hospital phase (Time 0). After 1 minute, the clamp was removed and the remaining tissue excised, resulting in a 25% lobectomy, consistent with a grade III liver injury. The hepatectomy specimen was weighed and divided by animal weight (kg) and reported as hepatectomy weight index (g/kg). Bleeding was spontaneous and removed continually via suction and measured by weight. Blood loss weight (g) was used to calculate estimated blood loss volume (%). At 15 minutes, animals were infused 250 mL normal saline (NS) over the next 60 minutes. This time point represents the arrival of a first responder (e.g. medic) who has limited carrying capacity. After 1 hour blood collection was discontinued and the abdomen packed then closed with towel clips.

After a total of 2 hours, the pre-hospital phase was completed and hospital arrival was simulated. This time point represents arrival of the patient to a hospital with surgical capabilities. The abdomen was reopened, residual blood suctioned, sponges collected, and blood loss quantified by weight. The liver injury was repaired with suture and the abdomen definitively closed. Invasive lines were removed and the carotid artery repaired. One unit of allogeneic whole blood (10 mL/kg) (Thomas Morris, Reisterstown, MD) was administered for hemoglobin <7 g/dL, otherwise animals received additional NS at 10 mL/kg. Anesthesia was stopped and the animals extubated.

### Experimental Group

Animals in the FTY720 group were treated 15 min after liver injury. FTY720 (0.3 mg/kg) (Cayman, Ann Arbor, MI) was diluted in 250 mL NS and administered over 60 min in place of the vehicle control infusion (250 mL NS).

### Post-surgical care and Histology

Animals were allowed to eat a regular diet and drink water following surgery. Buprenorphine (0.05–0.1 mg/kg IM/IV) was administered every 6–12 hours if the animals demonstrated any sign of pain. If the animals showed any sign of severe disability (e.g. inability to ambulate, eat, drink), severe infection, or uncontrolled pain, they were euthanized (Euthasol, Virbac AH, Fort Worth, TX) and taken for necropsy. At the end of the survival period (72 hours post-injury), surviving animals were euthanized and taken for necropsy. Samples of the heart, lung, liver, kidney, small intestine, mesenteric lymph node, and spleen were taken. A portion of each sample was fixed in 10% neutral buffered formalin. The formalin-fixed tissues were processed routinely, paraffin embedded, sectioned at approximately 5 micron, mounted on a glass slide and stained with Hematoxylin and Eosin (H&E) for histological evaluation. The remaining portion was stored in RNALater (Qiagen, Valencia, CA) or flash frozen using liquid nitrogen.

### Laboratory Data

Blood samples were collected at 0, 15, 30, 60, 90, and 120 minutes during the hemorrhage period and at 18, 24, 36, 48, and 72 hours during the survival period. Complete blood counts with differential were obtained using an automated cell counter (NDvia120 Hematology System, Siemens, Deerfield, IL).

### Immunohistochemistry

Immunohistochemical detection of CD3^+^ lymphocytes was performed on sections of formalin fixed, paraffin embedded blocks of lymph node and spleen. Tissue slides were deparaffinized using xylene followed by graded baths of ethanol. A DAKO Autostainer Plus Universal Staining System (DAKO, Carpenteria, CA) was used for automated immunohistochemical staining. Antigen retrieval was performed using Trilogy (Cell Marque, Rocklin, CA) for 30 minutes. Rat, anti- human CD3, monoclonal antibody, clone CD3–12, (Serotec, Raleigh, NC) was used at a dilution of 1∶50 and incubated overnight at 4C. Biotinylated goat anti-rat IgG specific polyclonal antibody (BD Biosciences, San Jose, CA) was applied as a secondary antibody at a 1∶100 dilution for 30 minutes at room temperature. The chromogen applied was 3, 3′ Diaminobenzidine (DAKO, Carpenteria, CA) for 10 minutes. The sections were counterstained with Hematoxylin (DAKO, Carpenteria, CA). External negative controls were processed identically as anti-CD3 but the primary antibody was substituted with normal rat serum. A tissue sample was considered positive if reactive cells, lymphocytes, demonstrated reactivity. An Automated Cellular Imaging System (ACIS, DAKO, Carpenteria, CA) was used to quantify the anti-CD3 immunohistochemistry staining.

Immunohistochemical detection of MPO was performed on sections of formalin fixed, paraffin embedded blocks of lung tissue. Antigen retrieval was performed using Proteinase K (DAKO, Carpenteria CA) for 30 minutes. Polyclonal rabbit, anti- human MPO (DAKO, Carpenteria CA) was used at a ready to use dilution and incubated at room temperature for 30 minutes. Envision, anti-rabbit (DAKO, Carpenteria CA) was applied as a secondary antibody for 30 minutes at room temperature. The chromogen applied was 3, 3′ Diaminobenzidine (DAKO, Carpenteria, CA) for 10 minutes. The sections were counterstained with Hematoxylin (DAKO, Carpenteria, CA). Normal serum controls were processed identically as with MPO but the primary antibody was substituted with normal rabbit serum. A tissue sample was considered positive if reactive cells, neutrophils, demonstrated intracytoplasmic reactivity in conjunction with a segmented nucleus. Neutrophils were noted mainly in circulation in small septal vessels and in large vessels. Cells with appropriate nuclear morphology and the presence of light to intensely reactive granules were counted; 5 random 40× fields were assessed.

### RNA Extraction and Quantitative Real-Time Polymerase Chain Reaction

Total RNA was isolated from liver tissue taken at necropsy using Qiagen RNeasy Mini Kit (Qiagen Inc. Valencia, CA) according to manufacturer's instructions. RNA purity and quantity were assessed by measuring the A_260_, A_280_, and A_230_on a Nanodrop Spectrophotometer (NanoDrop Technologies Inc. Wilmington, DE). RNA quality was determined from the 28S/18S rRNA ratio and RNA Integrity Number (RIN) using an Agilent 2100 BioAnalyzer (Agilent Technologies Inc. Santa Clara, CA). RIN values for all specimens in this study were ≥6.5. Satisfactory mRNA was available for FTY720 (n = 7) and the vehicle control (n = 5) groups. Reverse transcriptions were performed using Roche 1^st^ Strand Synthesis kits (Roche Diagnostics Corporation, Indianapolis, IN) according to the manufacturer's protocol. Quantitative real-time polymerase chain reaction (QRT-PCR) was performed using the 7900HT Fast Real-Time PCR System (Applied Biosystems, Foster City, CA) to assess mRNA transcript expression of 21 immune-related genes. Taqman chemistry was used. Primers for 18S rRNA target were used as an internal control for each reaction. Primers and probes for the targets of interest were obtained from Applied Biosystems (Table 1). All samples were run in duplicate. Individual samples were compared to averaged control tissue expression. Transcript quantification was derived using the comparative threshold cycle method and reported as the mean *n*-fold difference of the experimental sample to the control pool.

**Table 1:** RT-PCR targets with primer and probe sequences.

Target	Forward Primer	Reverse Primer	Probe
**IL-1α**	CTGAAGAAGAGACGGTTGAGTTTAAA	AAGTTGTATTTCATGTTGCTCTGGAA	CAGAAGAAGAAATCATCAAGCCCAGATCAGC
**IL-1β**	TTGAATTCGAGTCTGCCCTGT	CCCAGGAAGACGGGCTTT	CAACTGGTACATCAGCACCTCTCAAGCAGAA
**IL-6**	AATGTCGAGGCTGTGCAGATT	TGGTGGCTTTGTCTGGATTCT	AGCACTGATCCAGACCCTGAGGCAAA
**TNFα**	TGGCCCCTTGAGCATCA	CGGGCTTATCTGAGGTTTGAGA	CCCTCTGGCCCAAGGACTCAGATCA
**EDN-1**	CCTGCTCTTCCCTGATGGATAA	GGACAATGTGTTCTGGAGTGTTG	AGTGTGTCTACTTCTGCCACCTGGACATCA
**INOS**	CGTTATGCCACCAACAATGG	AGACCCGGAAGTCGTGCTT	ATCAGGTCGGCCATCACCGTG
**HIF-1α**	GCATTAAAGTTAGAACCAAATCCAGAGT	AAGGACTAGCTGGCTGATCTTGA	CTGGAACTTTCTTTTACCATGCCCCAGATT
**HSP70**	ACCATCGAGGAGGTGGATTAGA	ATGAAGTGATACAGAACAGAACTAAAATACAG	CCTCTTTGAGACCCAAGGACTTTGACATTGT
**TGF-β1**	AGGGCTACCATGCCAATTTCT	CGGGTTGTGCTGGTTGTACA	CCTAGACACTCAGTACAGCAAGGTCCTGGC
**C3**	CAAGAAATGATTGGTGGCTTCAA	GACCTGTGGTTCACAGATGTCTTT	AGCCTTTGTTCTCATCGCGCTGCA
**TGFα**	CCCAGATTCCCACAGTCAGTTC	AGCGTGCGCCGACGTA	AAGCCAGCATGTGTCTGCCACTCTG
**VCAM**	CTGTTCAAGTTGCTCCCAGGGAT	GAGCAGGTCATGTTCACAGAACTG	CGACCATCTCCGTCAACCCCTCC
**IL-2**	ACAGTTGCTTTTGAAGGAAGTTAAGAA	CCTGCTTGGGCATGTAAAATTT	CGAGAATGCTGATCTCTCCAGGATGCTC
**CD154**	GAAATGCACAAAGGCGATCA	TGCTGTTTTACTACTGGCCTCG	ATCCTCAAATTGCGGCACATGTCATAAG
**BAX**	CTCAAGCGCATTGGAGATGA	GTCCACGGCTGCGATCA	TCCTCTGCAGCTCCATGTTACTGTCCAG
**BCL-2**	TTCTACTTTGTCAGGCTTATGAAGGTT	TGTCCTTTGTCCCATAATAATTTTTTT	CAGGTCTTTATTGCTACTCAATTTGAATCTTCCCA
**FAS**	AATGGTATCGAAGAAACCAAAATAGAC	CGGAGCAGCTGGACTTTCTG	CATGCATGACAATCCCAAAGATACAGCTGA
**FAS-L**	GGCAAGCCTAACTCAAGATCCA	CATATACTTCACCCCAGAGACCAA	CAATTCCATAGGTGTCTTCCCATTCCAGAGG
**NF-κB**	CTGGCAGCTCTCCTCAAAGC	CACGAGTCATCCAGGTCATACAG	CTCAAAGTTCTCCACCAGGGGATCTGCTC
**COX-2**	CTGAACACCTCCGCTTTGC	AAGCACATCGCACACTCTATTATGT	CAGACCAGGCACCAGACCAAAGACCTC
**PPLA-2**	CTACTGTGGCCTAGGTGGATCAG	TCTCTGTAGCAGTTGTCGTGTGTCT	CGCAGCACCTGTCCAGTTCATCCAC

### Statistical Analysis

Animals were randomly assigned to the vehicle control or experimental FTY720 group. Survival analysis between groups was performed by Kaplan Meier survival plots with the log rank test. Continuous data was compared with the Student *t*-test or nonparametric tests as appropriate. Multiple comparisons were performed with one-way ANOVA. A one-way ANOVA with repeated measures design was used for continuous time-dependent comparisons. Statistical analysis was performed using SPSS (SPSS Inc., Chicago, IL). A two-tailed p value <0.05 was considered statistically significant. All data is represented as means ± standard error of the mean (SEM) unless otherwise specified.

## Results

**Figure 2:**
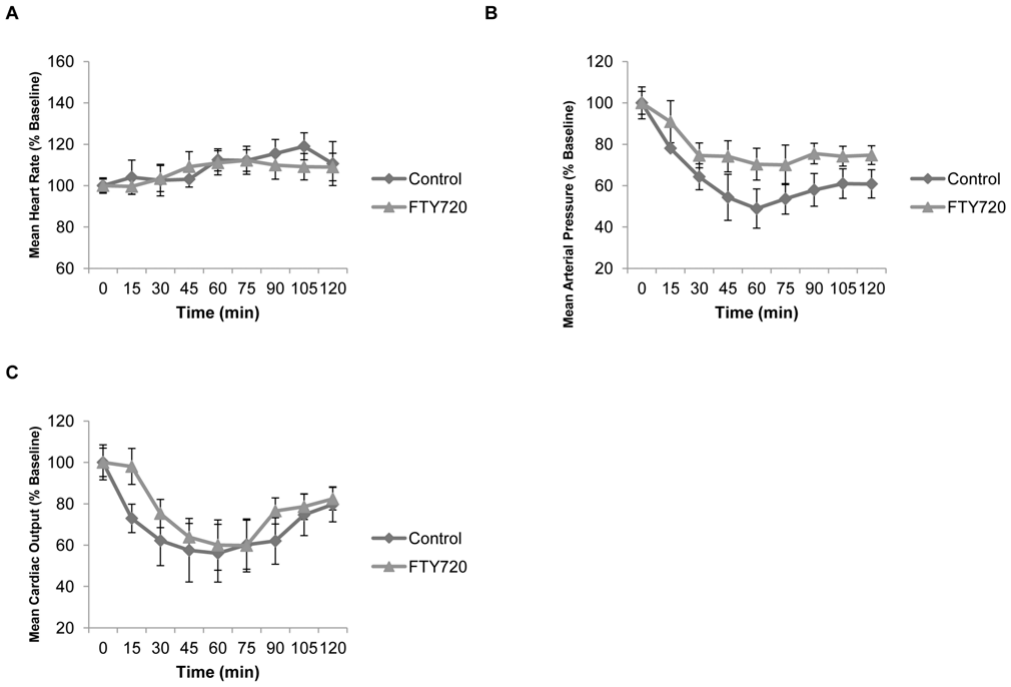
Hemodynamic profiles during hemorrhage period for FTY720 and vehicle control (in this and subsequent figures control is used for vehicle control) groups. **a**) Heart rate. **b**) Mean arterial pressure. **c**) Cardiac output. There were no statistical differences between the control and experimental hemodynamic profiles.

We used a well-validated and clinically relevant swine liver injury and resuscitation protocol with a 72 hour observation period to simulate hemorrhagic shock [13], [14]. Treatment animals were administered FTY720 fifteen minutes following liver injury. Both treatment and control groups underwent a standardized resuscitation protocol. Animal characteristics were comparable between vehicle control and the FTY720 treatment group (Table 2). Despite similar liver injury delivery, percent mean blood loss volume was lower in the FTY720 group compared to vehicle control but this was not statistically significant. In addition, blood transfusion requirements were identical between vehicle control and FTY720 groups. Importantly, invasive hemodynamic monitoring during the hemorrhage period demonstrated shock physiology in all animal groups (Figure 2). Both vehicle control and the FTY720 experimental group demonstrated a significant reduction in mean arterial pressure and cardiac output with elevation in heart rate compared to baseline. There were no statistical differences between the vehicle control and experimental hemodynamic profiles.

**Table 2:** Animal clinical parameters.

Variable	Control (n = 9)	FTY720 (n = 9)	p-value
Mean animal weight [kg]	32.5±1.7	30.1±0.5	NS^1^
Animal gender - M∶F	5∶4	5∶4	NS^2^
Mean hepatectomy weight index [g/kg]	0.31±0.04	0.37±0.04	NS^1^
Mean blood loss volume %	46.8±3.4	34.3±4.9	NS^1^
Transfusion requirement - no. (%)	7 (78)	7 (78)	NS^2^

1 Student *t*-test;

2 chi-squared.

**Figure 3:**
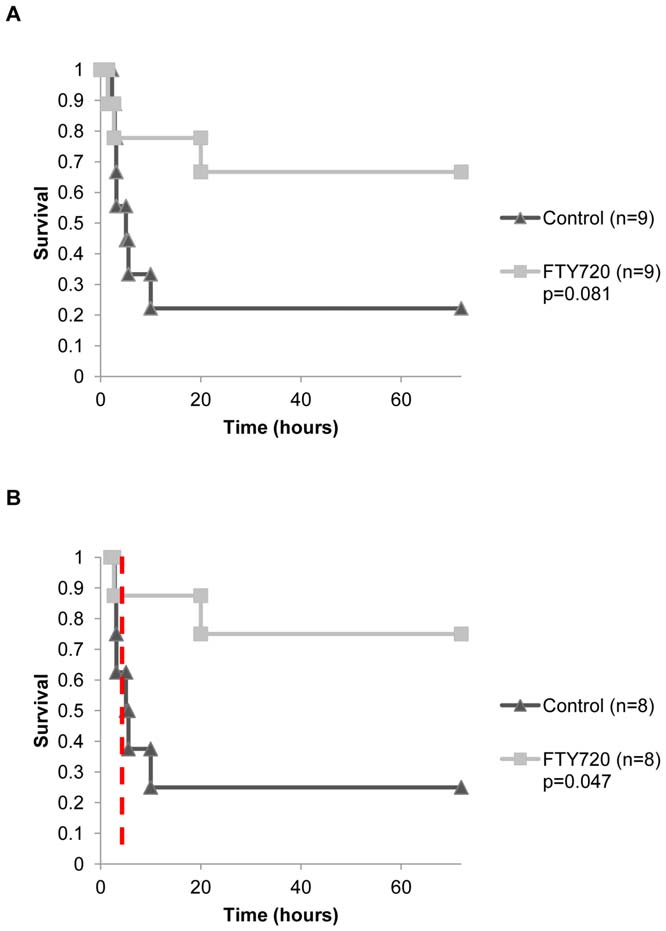
Kaplan-Meier survival curve for experimental groups versus control with log-rank test for statistical comparison. **a**) Overall survival. **b**) Reperfusion period survival. Dashed red line indicates end of hemorrhage and beginning of reperfusion period.

Overall survival in the FTY720 experimental group was improved compared to control although not statistically significant (Figure 3A). The overall survival was 22.2% (2/9) in the vehicle control and 66.7% (6/9) in the FTY720 group (p = 0.081). One animal in both the FTY720 and vehicle control group died during the hemorrhage period and were not included in the reperfusion period (following hemorrhage) survival analysis. During the reperfusion period survival was statistically improved in the FTY720 group compared to control (Figure 3B). The reperfusion survival was 25% (2/8) in the vehicle control and 75% (6/8) in the FTY720 group (p = 0.047). Thereby, disruption of the lymphocyte response to shock during hemorrhage appeared to significantly reduce mortality associated with reperfusion injury.

**Figure 4:**
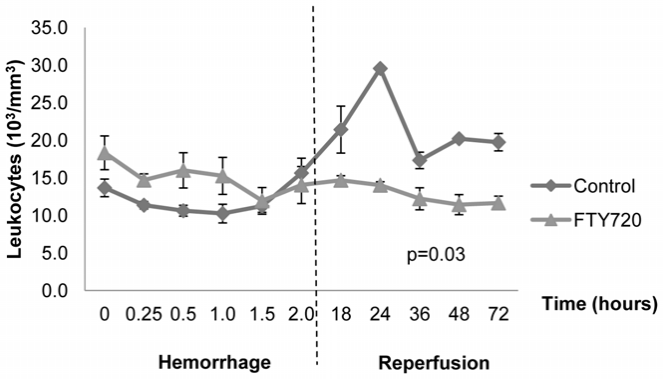
Peripheral leukocyte counts during hemorrhage and reperfusion periods. Dashed line indicates end of hemorrhage and beginning of reperfusion period. Leukocytes were significantly decreased during the reperfusion period in the FTY720 group (p = 0.03). Data is depicted as mean ± SEM.

**Figure 5:**
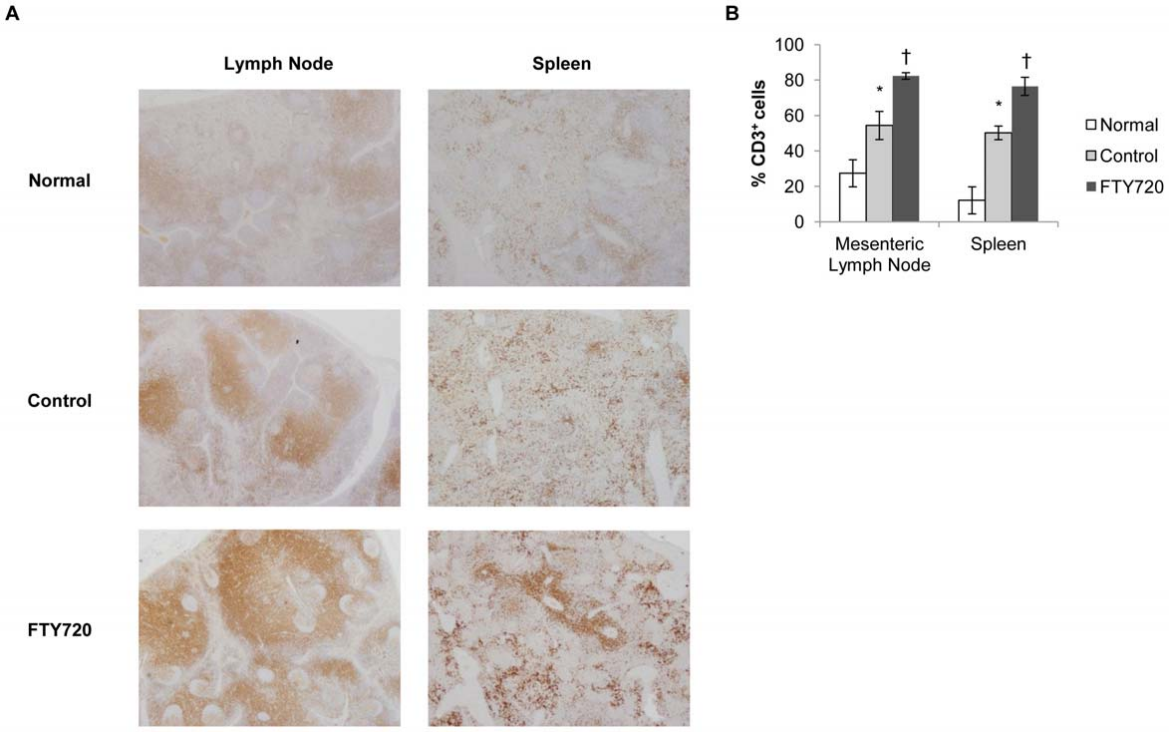
Central lymphocyte counts at time of necropsy. **a**) Representative immunohistochemistry pictures (20× magnification) of anti-CD3 staining of normal, control, and FTY720 mesenteric lymph node and spleen tissue. **b**) Quantification of anti-CD3 reactivity by ACIS. * indicates p<0.05 compared to normal, unmanipulated tissue; † indicates p<0.05 compared to control. Data is depicted as mean ± SEM.

Total peripheral, or circulating, leukocytes were not different during hemorrhage, but were significantly reduced during the reperfusion period in the FTY720 group (Figure 4). We used anti-CD3 immunohistochemistry staining of mesenteric lymph nodes and spleen tissue at time of necropsy to evaluate central lymphocyte sequestration (Figure 5). There was significant central CD3^+^ T lymphocyte sequestration in the FTY720 group compared to control in both mesenteric lymph node and spleen (Figure 5A). Interestingly, significant central CD3^+^ T lymphocyte sequestration was evident in the vehicle control group compared to naïve swine mesenteric lymph node and spleen tissue (Figure 5B). This finding suggests that some degree of T lymphocyte sequestration is part of the normal response to hemorrhagic shock. Importantly, FTY720 mediated peripheral leukocyte reduction with central lymphocyte sequestration and conferred a survival advantage in this liver injury model.

**Figure 6:**
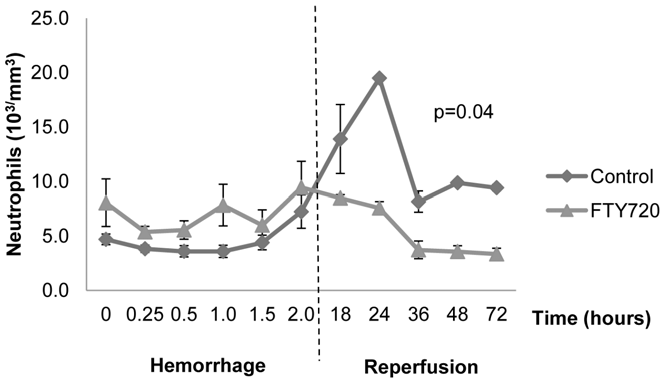
Peripheral neutrophil counts during hemorrhage and reperfusion periods. Dashed line indicates end of hemorrhage and beginning of reperfusion period. Data is depicted as mean ± SEM.

**Figure 7:**
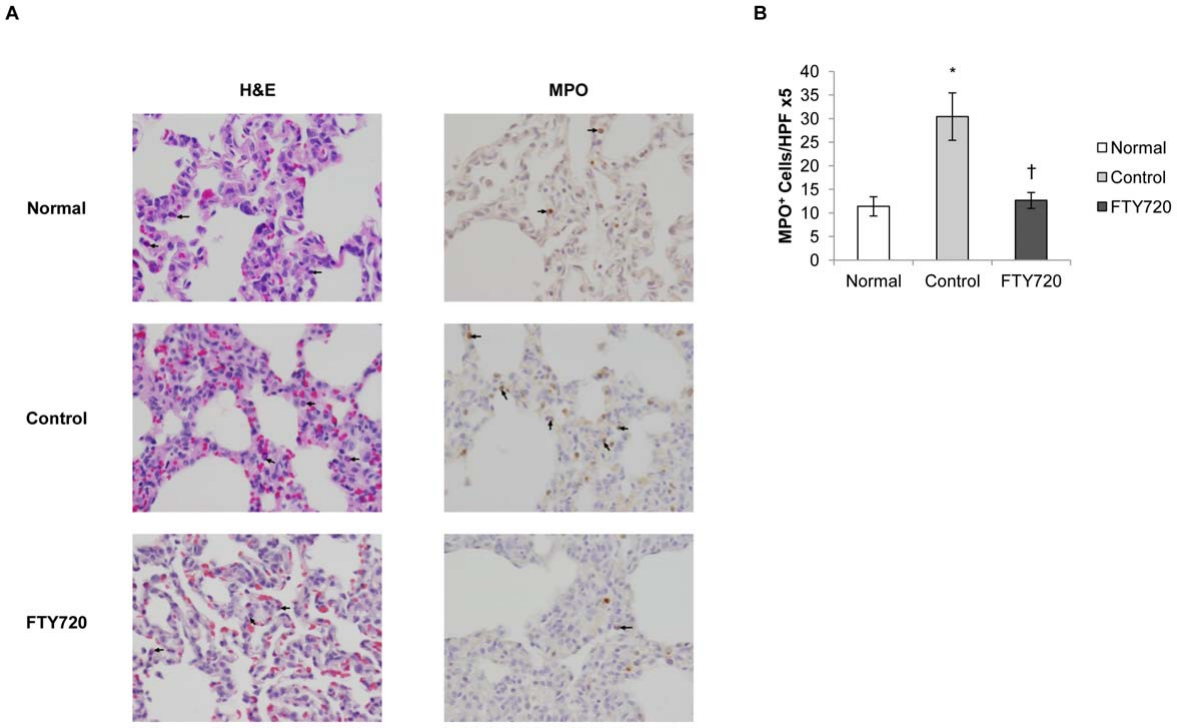
Lung tissue neutrophil infiltration at time of necropsy. **a**) Representative histology pictures (400× magnification) of normal, control and PATG tissue. **b**) Quantification of anti-MPO reactivity. * indicates p<0.05 compared to normal, unmanipulated tissue. † indicates p<0.05 compared to control group. Data is depicted as mean ± SEM.

In order to study the innate cellular response to hemorrhagic shock, we examined peripheral blood and tissue neutrophil levels in the FTY720 and control groups. Neutrophils have been established as mediators of injury following hemorrhage in both animal and human studies [15], [16], [17]. During reperfusion, peripheral blood neutrophil numbers were markedly increased in the vehicle control group (Figure 6). This peripheral neutrophil response was attenuated in the FTY720 group throughout the reperfusion period. The tissue neutrophil response to hemorrhagic shock was evaluated by examining lung tissue neutrophil infiltration, a major target organ of neutrophils following shock [18], [19]. Neutrophil infiltration (H&E stained sections and immunohistochemistry using anti-MPO antibody) in lung tissue was significantly increased in vehicle control animals when compared to naïve lung tissue (Figure 7). In contrast, lung tissue neutrophils were significantly decreased in the FTY720 group compared to control. These findings indicate that both the peripheral blood and tissue neutrophil response was attenuated in the FTY720 group, suggesting that lymphocyte manipulation also disrupted the innate neutrophil response to hemorrhagic shock.

**Figure 8:**
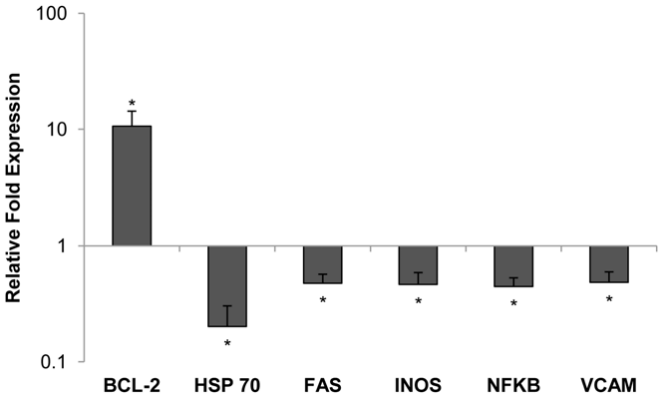
Inflammatory gene transcript expression in liver tissue of FTY720 relative to the control group. Mean value of relative fold expression is depicted on a logarithmic scale. * indicates p<0.05 compared to control group.

In order to evaluate the innate molecular response to hemorrhagic shock, we analyzed gene transcripts of 21 inflammatory targets associated with IRI from liver tissue, a major effector organ of the systemic immune response to shock (Table 1) [6]. Expression of six gene targets were statistically different from the control group when compared to the FTY720 group; the anti-apoptotic gene BCL-2 was up-regulated and the apoptotic gene FAS was down regulated; innate immunity genes HSP70, INOS, and NFκB were down-regulated; the adhesion gene VCAM was also down-regulated compared to vehicle control animals (Figure 8). Thus, lymphocyte modulation also appeared to reduce apoptosisand liver inflammation at the molecular level following hemorrhagic shock.

## Discussion

In this study, we show that lymphocyte modulation with FTY720 significantly improves reperfusion survival following experimental hemorrhagic shock in a clinically relevant large animal model. T lymphocyte sequestration appears to modulate innate cellular and molecular activation following hemorrhagic shock. Specifically, disruption of the innate lymphocyte response resulted in significantly decreased circulating and lung tissue infiltrating neutrophils and decreased expression of liver immune-related genes. T lymphocytes mediate critical innate events following hemorrhage, and lymphocyte modulation may ameliorate reperfusion injury.

The immune system is designed to respond to local injury and is necessary for hemostasis, protection against microorganism invasion, and initiation of tissue repair. Severe trauma and hemorrhagic shock induce a systemic inflammatory response that results in inappropriate immune activation and secondary injury to the host. Severe injury provokes the simultaneous activation of potent molecular and cellular components of innate immunity, including complement, cytokines, chemokines, neutrophils, and monocytes [20]. These innate events culminate with organ injury to the host, manifesting clinically as the systemic inflammatory response syndrome, acute respiratory distress syndrome, and ultimately multi-organ dysfunction syndrome [1]. Modulation of the immunologic response to injury is a major therapeutic goal to reduce associated morbidity and mortality.

The majority of research on clinical therapeutics for ischemia reperfusion injury has focused on monocyte and neutrophil adhesion blockade. However, despite promising preclinical data, results of phase 2 and 3 trials of neutrophil anti-adhesion therapy in ischemia-reperfusion disorders have been disappointing [21]. In two clinical trials testing humanized CD18 monoclonal antibodies in the setting of traumatic injury, mortality and other primary end points were not significantly affected [22], [23]. These failures are likely the result of the redundancy of adhesion pathways, but also suggest that the neutrophil is not central in the innate immune response to IRI.

Adaptive lymphocyte activation is conventionally described in the presence of foreign antigen bound to self-MHC molecules together with antigen-presenting cell costimulation signals. However, there is emerging evidence that lymphocytes are rapidly activated in an alloantigen-independent manner in the setting of IRI [24] [9]. Danger signals released in IRI may activate lymphocytes and lead to innate function prior to classical adaptive function [25]. Furthermore, hypoxia may be sufficient for lymphocyte activation, as CD4^+^ T lymphocytes have been shown to increase adhesion to endothelial monolayers following anoxia modulation [26], [27]. The first direct proof of the role for T lymphocytes in IRI was in a mouse liver ischemia model where a marked preservation of liver function was found in T cell deficient athymic (*nu/nu*) mice compared to wild type mice [7]. This was associated with a decrease in neutrophil infiltration, which was restored with adoptive transfer of wild type T cells into the athymic mice. The role of T lymphocytes as critical cellular mediators of the innate immune response to IRI has been substantiated in multiple subsequent IRI models [6], [7], [8], [28], [29]. Altogether, these pre-clinical studies demonstrate a novel, innate function of T lymphocytes in the setting of ischemic injury.

The innate role of T lymphocytes offers the potential for targeting lymphocytes in the setting of severe injury and hemorrhagic shock. FTY720 is a recently introduced immunomodulator that sequestrates T lymphocytes to secondary lymphoid organs and reduces circulatory lymphocytes by targeting receptors for sphingosine 1-phosphate. Specifically, FTY720 engages four of the five known S1P receptors (S1P_1_, S1P_3_, S1P_4_, and S1P_5_). In lymph nodes FTY720 acts as an agonist of the S1P_1_ receptor resulting in functional antagonism and internalization of S1P_1_ receptors on lymph-node T cells [30], [31]. FTY720 has been shown to ameliorate IRI in various animal models and T lymphocyte modulation has been mechanistically implicated [32]. In a rat renal transplant model, FTY720 treatment improved renal function and reduced intragraft neutrophil infiltration despite no change in adhesion molecules expression, highlighting the central role of the T lymphocyte in IRI [33]. In a series of pilot experiments using our porcine HS IRI model, we have demonstrated the ability of T cell depletion using porcine anti-thymocyte globulin (PATG) to produce the same effect as described in this report further highlighting the central role of T cells in hemorrhagic shock [34]. The overall survival was 22% (2/9) in the control and 75% (6/8) in the PATG groups, p = 0.09; during the reperfusion period (following hemorrhage) survival was 25% (2/8) in the control and 100% (6/6) in the PATG groups, p = 0.008. Results from these studies suggest that the disruption of the innate lymphocyte response to hemorrhagic shock appeared to significantly reduce mortality associated with reperfusion injury. However, these studies were limited by the need to pre-treat animals and therefore only served as proof of concept. Here we corroborate these findings in demonstrating that T lymphocyte modulation following hemorrhage with FTY720 reduces innate immune-mediated injury during reperfusion.

Neutrophils have been established as mediators of injury following hemorrhage in both animal and human studies [15], [16], [17]. In particular, neutrophils have been shown to mediate acute lung injury following hemorrhagic shock, a significant contributor to morbidity and mortality after injury [18], [19]. Interestingly, T lymphocytes may coordinate innate neutrophil action following ischemic injury. In an intestinal IRI model, T lymphocytes were shown to orchestrate neutrophil trafficking [6]. Thereby, by disrupting T lymphocyte function in the setting of hemorrhage, neutrophil action may also be affected. In the current study, T lymphocyte depletion resulted in significant circulating and tissue infiltration neutrophil inhibition. The circulating neutrophil response was diminished during the reperfusion period in FTY720 treated animals. In addition, lung neutrophil infiltration was also significantly attenuated during reperfusion in the lymphocyte depletion group. The reduction in lung tissue neutrophil infiltration likely contributed to the improved survival in the FTY720 treated animals. In sum, innate lymphocyte and neutrophil function are intimately linked, and lymphocyte disruption in the setting of hemorrhage appears to inhibit neutrophil mediated immune injury.

Several important limitations of this study must be addressed. It is noteworthy that the FTY720 treated animals appeared to lose less blood than controls, although the difference was not statistically different and all animals demonstrated shock physiology after hemorrhage. Sphingosine 1-phosphate receptors are present on vascular endothelium in addition to secondary lymphatic organs, and have been shown to regulate vascular tone [35]. In particular, FTY720 has been shown to reduce portal pressure in a small animal model as well as stabilize pulmonary and vascular endothelium [36]. Additionally, S1P places a role in platelet aggregation suggesting the possibility of T lymphocyte independent effects [37]. One possible role of these non T lymphocyte mediated effects in hemorrhagic shock is that FTY720 activation of sphingosine 1-phosphate receptors on mesenteric vascular endothelium results in relative splanchnic vasoconstriction, resulting in decreased blood loss during visceral hemorrhage. Although these findings confound the current experiment, it is clearly a beneficial side effect of the therapeutic employment of FTY720 for intra-abdominal hemorrhagic shock. FTY720 has also been associated with significant bradycardia in clinical trials, also related to sphingosine 1-phosphate receptors on cardiomyocytes [38]. While not observed in the current experiment, drug induced bradycardia would have obvious detrimental effects during hemorrhage. Finally, while the role of the lymphocyte in early innate immune activation is under intense investigation, the precise mechanisms of lymphocyte mediated ischemia-reperfusion injury have not been fully elucidated. Thereby, a complete biological explanation for the survival advantage conferred by lymphocyte disruption following hemorrhage is not yet available but the ability of T cell manipulation by either sequestration or depletion to ameliorate hemorrhagic shock suggest that this is primarily a T lymphocyte and not a FTY720 mediated effect. In order to fully address these questions future mechanistic studies are warranted to clarify the role of lymphocytes in immune injury following hemorrhage.

The current experiment demonstrates the novel use of a lymphocyte modulator administered following hemorrhage in a clinically relevant animal model. This lends to direct clinical translation in which a lymphocyte therapeutic, such as FTY720, can be administered during resuscitation of a trauma patient. In addition, there is a large array of pharmacologic strategies currently available that target lymphocyte function, primarily for clinical use in the setting of transplantation and autoimmune diseases. Humanized monoclonal antibodies, such as anti-CD52 mAb (Alemtuzumab), are well tolerated and may be clinically applicable following significant hemorrhage or injury [39]. Importantly, the identification of lymphocytes as important components of the innate immune system may lead to harnessing advancements in lymphocyte biology for novel hemorrhagic shock therapies. We are currently exploring these potential therapeutic pathways.

In conclusion, we show that lymphocyte sequestration with FTY720 significantly improves reperfusion survival following experimental hemorrhagic shock in a large animal model. Lymphocyte immunomodulation appears to attenuate innate cellular and molecular activation following hemorrhagic shock. Specifically, disruption of the innate lymphocyte response resulted in significantly decreased circulating and lung tissue infiltrating neutrophils and decreased expression of liver inflammatory genes. In conclusion, lymphocyte sequestration with FTY720 offers a novel and clinically applicable approach towards abrogating immune mediated reperfusion injury in trauma and surgical patients.
